# Adolescent behavioural risk screening in primary care: physician’s point of view

**DOI:** 10.1093/fampra/cmad106

**Published:** 2023-11-16

**Authors:** Taslina Eisner-Fellay, Joan-Carles Suris, Yara Barrense-Dias

**Affiliations:** Research Group on Adolescent Health, Department of Epidemiology and Health Services, Center for Primary Care and Public Health (Unisanté), University of Lausanne, Lausanne, Switzerland; Research Group on Adolescent Health, Department of Epidemiology and Health Services, Center for Primary Care and Public Health (Unisanté), University of Lausanne, Lausanne, Switzerland; Research Group on Adolescent Health, Department of Epidemiology and Health Services, Center for Primary Care and Public Health (Unisanté), University of Lausanne, Lausanne, Switzerland

**Keywords:** adolescent health, confidentiality, health risk behaviours, physicians, prevention, primary health care

## Abstract

**Background:**

Despite regular consultation between adolescents/young adults (AYA) and their physicians, they are not regularly screened for psychosocial risk behaviours. This study examines physicians’ self-reported psychosocial risk behaviour screening in AYA. It aims to highlight which elements hinder or improve screening abilities.

**Methodology:**

The design was a cross-sectional quantitative survey. Data were obtained through a self-reported questionnaire sent out to primary care physicians (PCP) in Switzerland in 2018. The target population consisted of 1,824 PCP (29% response rate). Participants were asked whether they screened youths from 3 age groups [10–14 y/o, 15–20 y/o, and 21–25y/o] for the HEEADSSS items during child well visits and routine checkups. Barriers to screening included primary consultation motive prioritization, insufficient time, patient compliance, reimbursement, lack of skills related to adolescent health, lack of referral options. Data were analysed first through a bivariate analysis using Chi-square tests then through a multinomial logistic regression.

**Results:**

The majority of physicians partook in preventive screening for 3–5 psychosocial risk elements. They reported the primary consultation motive as well as a lack of available time as having a high impact on their screening habits. Physician’s experience and having discussed confidentiality were related to an increase in the number of topics addressed. Confidentiality remained a significant variable throughout all analyses.

**Conclusion:**

Barriers such as lack of consultation time and prioritization issues were found by physicians to be critical but did not hinder screening habits. The main element impacting screening habits was assuring confidentiality and the second is self-efficacy.

**Key messages**

- Most physicians screen youths for 3–5 psychosocial risk behaviours.
- Barriers to screening such as lack of time were not found to have an impact.
- The main element impacting screening habits was assuring confidentiality.
- The second element was physician’s self-efficacy regarding adolescent health.

## Background

Adolescence sees the development of new psychosocial areas of concern, such as mental health issues, drug use or at-risk sexual behaviors.^1^ These issues may impact the development of adolescents and their transition to adulthood.^2,3^ They have previously been linked to self-harm, which is the second cause of premature death in youths in Europe, after road-related deaths.^4^ Interventions that include primary care professionals training have been shown to improve psychosocial risk factor screening,^5^ which, in turn, has been shown to improve risk factor detection as well as health outcomes.^6,7^ During such training, screening tools developed to aid professionals are often addressed. One such tool is the HEEADSSS assessment guide (Home, Education/employment, Eating, peer-related Activities, Drugs, Sexuality, Suicide/mental health, and Safety from injury and violence),^8^ which allows for an evaluation of the youth’s social, educational, and home environment. It recommends assuring confidentiality as an opening statement before engaging with screening. This tool offers an opportunity to detect issues related to these different domains at an early stage, enabling timely and appropriate interventions. Primary prevention is thought to be superior to secondary or tertiary prevention regarding health impact since it allows for anticipatory guidance.^9^

Previous studies, based both in Switzerland and in the United States,^10,11^ have shown that although adolescents regularly consult their primary care provider (PCP), they are not routinely screened for psychosocial risk behaviours such as alcohol or drug consumption, eating issues, or suicidal behaviours.^12,13^ Barriers to behavioural risk screening have previously been established to be insufficient financial compensation, insufficient consulting time, or lack of resources and this is independent of the health system examined.^14,15^

This study examines physicians’ self-reported psychosocial risk behaviours screening in adolescents and young adults. It aims to highlight which elements hinder or improve screening abilities to identify leads to increased screening habits. It will, therefore, first evaluate to what degree the different HEEADSSS topics are screened for, and which physician’s sociodemographic characteristics are associated to this screening. Second, it will evaluate which barriers to screening are rated as important, and whether they impact screening habits.

## Methods

### Study design

The study was a cross-sectional survey with a quantitative methodology.

### Setting/subjects

The data were collected in Switzerland from February to October 2018. The target population consisted of 1,824 physicians with a primary care specialization (paediatricians or general practitioners (GP)) whose practice was based in the French-speaking part of Switzerland (2 140 124 inhabitants in 2019). Participants were recruited based on a mix of purposive and volunteer sampling methods. First, physicians were invited to participate in the survey via their postal or email addresses when known (*N* = 954). Second, to prevent under-coverage since known postal addresses were limited geographically, medical societies were asked to transfer the survey to their members or post it on their web pages. All in all, 525 participants answered the survey. Participants who had incomplete answers (*N* = 37) or did not consult with youths aged 10–25 (*N* = 32) were excluded, leading to a final sample of 456 participants. Physicians with a second specialization such as gynaecologist, surgeons, or psychiatrist were included when available since they can act as PCP for certain specific youths.

### Data collection

#### Instrument

Data were obtained through a self-administered questionnaire comprised of 12 close-ended questions developed for the purpose of this study. The questions were directed to child well visits and routine checkups, as physicians were asked to set aside their screening habits regarding emergency consultations. It consisted of questions from the following topics: demographics, specialization, frequency of addressing the HEEADSSS topics, experience regarding adolescent health and importance of barriers to screening. Questions regarding HEEADSSS topics’ formulation were broad (e.g. during consultations do you have the opportunity to address alcohol consumption? With 5 possible responses ranging from never to always). The barriers’ selection was based on preexisting literature.^16–18^ The questionnaire was previously tested on a sample of PCP prior to the recruitment, allowing for clarification when necessary.

### Independent variables

Sociodemographic variables included were: physician’s gender, age (under 40 y/o, 40–49 y/o, 50 y/o, and over), and specialty (paediatrician, general practitioner, other).

Physician’s own experience regarding adolescent health: Physicians were asked to rate their experience on a scale from 1 (weak) to 5 (excellent). The answers were then recoded into 3 categories: inexperienced,^1,2^ experienced,^3^ very experienced.^4,5^

Discussing confidentiality: Physicians were asked to indicate whether they addressed confidentiality during consultations. The response categories were: always, only when necessary and never.^19^ Answers were then recoded into: Yes (‘always’ screened allowing for a primary prevention) and No (‘only when necessary’ or ‘never’, allowing for secondary/tertiary/no prevention). Confidentiality was included as an explanatory variable since we postulated it is one of the keystone elements to discussing sensitive topics with youths.^20^

Barriers to screening included prioritization [emergency of primary consultation motive], insufficient consultation time, barriers related to family or patient compliance, reimbursement issues, lack of skills regarding adolescent health, lack of referral options. For each item, a five-point Likert scale was used (1 = no impact at all, 2 = low impact, 3 = medium impact, 4 = high impact, 5 = very high impact). Answers were then recoded into 3 categories: no impact [1–2], medium impact [3], and high impact [4–5]. Reimbursement issues relate to the Swiss health system which is based on fee-for-service.

### Dependent variables

The 8 risk behaviours and environmental elements analysed were those included in the HEEADSSS screening method, namely: Home, Education, Eating (eating habits and body image), Activities (sport practice, screen use, hobbies), Drugs (tobacco, alcohol, marijuana, other drugs), Safety, Suicide/mental health (mood, emotions), and Sexuality. This screening tool has been largely validated^21^ and is the one mainly used in Switzerland.

Physicians rated their screening habits for youths categorized into three age groups (10–14y/o, 15–20 y/o and 21–25y/o). The questions for each one of the age groups were identical. They were comprised of 3 possible answers: always, only when necessary, and never.^19^ Answers were recoded into: Yes (‘always’ screened allowing for a primary prevention) and No (‘only when necessary’ or ‘never’, allowing for secondary/tertiary/no prevention).

The 8 HEEADSSS topics were added, obtaining a score ranging from 0 to 8, with a higher score indicating a more thorough screening habit.

As only 13% scored zero topics, and a majority of physicians screened for 3–5 topics, the score was clustered into 3 categories according to the number of topics screened for: none/few (0-1-2); average (3-4-5); above average (6-7-8).

### Data analysis

Data were analysed using Stata 14 (StataCorp, College Station, Texas). At the bivariate level, Chi-square tests were performed to evaluate whether a relation existed between the 3-category scale of risk behaviour screening and the previously mentioned explanatory variables. The results are presented as point prevalence. All statistically significant variables (*P* value < 0.05) at the bivariate level were then entered into a multinomial logistic regression to analyse the predictor factors impacting screening rates. Four models were created to assess whether all outcomes measured the same phenomenon. The first one included only confidentiality, the second one added physician’s experience, and the third one included the following sociodemographic variables when statistically significant: age, gender, and specialty. The last model added significant barriers to screening. The group screening for 3–5 of the HEADSSS items (i.e. determined to be an average number of items) was used as the reference category since it allowed us to determine which explanatory variables were associated with physicians’ screening for more or less than the average number of items. Only 4 paediatricians reported seeing youths aged 20–15 years of age. For that reason, they were added to “others” for that particular age category. The results are presented as relative risk ratios with 95% confidence intervals (95%CI).

All data were analysed independently according to the 3 youth age groups.

#### Ethics approval

Participation was voluntary and non-remunerated. Since this study is a survey evaluating physicians’ opinions without addressing personal or patient data, it falls under the ‘quality control and opinion research’ category and is thus not subjected to the Swiss human research act. This was confirmed by the Ethics committee of the canton of Vaud, Switzerland prior to the study beginning.

## Results

Out of the 456 participants, 58% were GP, 19% paediatricians, and 22% other specialists (66% psychiatrists, 19% gynaecologists, and 15% surgeons). Fifty-four percent of them were males, with 58% aged 50 or above (Table 1).

**Table 1.** Descriptive analysis of physicians located in the French speaking part of Switzerland in 2018 (*N* = 456) by physician’s gender, level of experience, topics discussed and barrier to screening’s importance.

Gender				
		Female (*N* = 208)	Male (*N* = 248)	Total (*N* = 456)
Specialty	Paediatrician	26.67	12.55	19.06
	GP	47.56	67.68	58.40
	Other	25.78	19.77	22.54
Age	<40 y/o	19.56	8.37	13.52
	40–49 y/o	36.89	21.29	28.48
	>50 y/o	43.56	70.34	57.99
Experience level	Inexperienced	22.22	30.04	26.43
	Experienced	41.33	38.78	39.96
	Very experienced	36.44	31.18	33.61
No. of topics addressed	0–2	13.94	30.65	23.03
	3–5	40.87	41.13	41.01
	6–8	45.19	28.23	35.96

Level of experience				
		Inexperienced (*N* = 120)	Experienced (*N* = 182)	Very experienced (*N* = 153)
Gender	Male	61.24	52.31	50.00
Age	<40 y/o	18.60	15.38	7.32
	40–49 y/o	30.23	30.77	24.39
	>50 y/o	51.16	53.85	68.29
Specialty	Paediatrician	3.10	18.97	31.71
	GP	67.44	64.62	43.90
	Other	29.46	16.41	24.39
Confidentiality by age group		40.76	59.56	39.39

HEEADSSS topics by age group				
HEAADSSS topics		10–14 y/o (*N* = 238)	15–20 y/o (*N* = 366)	21–25 y/o (*N* = 362)
Home	55.46	64.21	50.69
Education	79.83	77.87	69.70
Eating	51.26	50	49.59
Activities	71.85	66.12	64.46
Drugs	23.11	46.72	49.59
Safety	16.39	15.85	11.33
Suicide/mental health	56.30	48.91	44.63
Sexuality	24.79	39.07	28.93

Importance attributed to screening barrier				
		No impact (%)	Medium impact (%)	High impact (%)
Barriers to screening	Prioritization	22.59	24.56	52.85
	Consultation time	28.29	24.57	47.15
	Patient compliance	44.08	33.11	22.81
	Reimbursement	71.49	19.52	8.99
	Lack of skills	51.21	34.73	14.07
	Lack of referral options	60.66	27.69	11.65

There was no statistically significant difference regarding gender depending on the level of experience reported. However, physicians over the age of 50 felt more experienced (68%) compared to those younger than 40 (7%) and GPs reported feeling ‘very experienced’ the most (43%) when compared to pediatricians (31%), or other specialties (24%). Fifty-one percent of physicians consulted with youths aged 10–14 y/o, and 78% with youths aged 15–20 and 21–25 (Table 1).

When considering all patient age groups, most physicians (43%) partook in preventive screening for 3–5 topics of behavioural risks. The most prevalent topics discussed were education (80%) and activities (58%) for the 10–14 y/o; home (64%), education (78%), and activities (66%) for the 15–20 y/o and finally education (70%) and activities (65%) for the 21–25 y/o (Table 1).

Barriers reported as having a high impact regarding behavioural risk screening were prioritization (53%) as well as the lack of available consultation time (47%). Lack of referral options as well as lack of skills regarding adolescent health were reported as having no impact (61% and 51%, respectively) by a majority of respondents (Table 1).

In the bivariate analysis, female physicians tended to screen more thoroughly than their male counterparts regardless of patient age group. Paediatricians tended to screen for more topics when considering the 10–14 age group, and GP for the 15–20 age group. The results became non-significant for the 21–25 age group, which was mainly treated by GP (75%), and only 1% by paediatricians. Both physician’s experiences and having discussed confidentiality were related to an increase in the number of topics screened for, regardless of the patient age group.

The majority of barriers (prioritization, consultation time, patient compliance, referral options) held no significant relation regarding the number of topics addressed. Only the barrier associated with a lack of skills regarding adolescent health, rendered a significant result for the 15–20 and 21–25 age groups, with physicians who recognized that barrier as important screening for significantly less topics (Table 2).

**Table 2.** Results of bivariate analysis on the number of topics addressed by the physician according to independent variables and barriers to screening, presented as point prevalence, 3 sub-sections according to youth’s age groups, sample of 465 physicians located in the French speaking part of Switzerland in 2018.

10–14 y/o		0–2 topics (*N* = 63)	3–5 topics (*N* = 124)	6–8 topics (*N* = 51)	*P* value
Gender	Female	28.57	52.42	64.71	0.000
Age	<40	9.52	14.52	25.49	0.002
	40–49	23.81	30.65	19.61	
	>50	66.67	54.84	54.90	
Speciality	Paediatrician	9.52	45.16	49.02	0.000
	GP	73.02	38.71	35.29	
	Other	17.46	16.13	15.69	
Experience	Inexperienced	22.22	8.87	1.96	0.000
	Experienced	53.97	41.94	25.49	
	Very experienced	23.81	49.19	72.55	
Confidentiality	Discussed	12.70	39.52	78.43	0.000
Prioritization	No impact	12.70	22.13	28.00	0.316
	Medium impact	26.98	27.87	22.00	
	High impact	60.32	50.00	50.00	
Consultation time	No impact	23.81	21.31	36.00	0.115
	Medium impact	34.92	29.51	16.00	
	High impact	41.27	49.18	48.00	
Patient compliance	No impact	57.14	38.52	54.00	0.063
	Medium impact	23.81	36.89	20.00	
	High impact	19.05	24.59	26.00	
Reimbursement	No impact	76.19	77.87	70.00	0.721
	Medium impact	14.29	16.39	20.00	
	High impact	9.52	5.74	10.00	
Lack of skills	No impact	53.23	58.20	68.00	0.386
	Medium impact	35.48	29.51	28.00	
	High impact	11.29	12.30	4.00	
Lack of referral option	No impact	59.68	58.20	62.00	0.628
	Medium impact	35.48	30.33	28.00	
	High impact	4.84	11.48	10.00	

15–20 y/o		0–2 topics (*N* = 103)	3–5 topics (*N* = 140)	6–8 topics (*N* = 123)	*P*-value
Gender	Female	29.13	46.43	60.98	0.000
Age (y/o)	<40	10.68	13.57	16.26	0.319
	40–49	22.33	30.71	29.27	
	>50	66.99	55.71	54.47	
Speciality	Paediatrician	7.77	18.57	33.33	0.000
	GP	71.84	64.29	46.34	
	Other	20.39	17.14	20.33	
Experience	Inexperienced	25.24	20.71	8.13	0.000
	Experienced	53.40	42.14	33.33	
	Very experienced	21.36	37.14	58.54	
Confidentiality	Discussed	29.13	59.29	85.37	0.000
Prioritization	No impact	19.61	18.12	27.12	0.285
	Medium impact	22.55	28.26	27.12	
	High impact	57.84	53.62	45.76	
Consultation time	No impact	27.45	21.74	32.20	0.084
	Medium impact	32.35	23.19	22.03	
	High impact	40.20	55.07	45.76	
Patient compliance	No impact	53.92	39.86	44.92	0.164
	Medium impact	25.49	37.68	28.81	
	High impact	20.59	22.46	26.27	
Reimbursement	No impact	72.55	71.74	72.03	0.793
	Medium impact	17.65	21.01	16.95	
	High impact	9.80	7.25	11.02	
Lack of skills	No impact	49.50	49.28	68.64	0.005
	Medium impact	33.66	38.41	26.27	
	High impact	16.83	12.32	5.08	
Lack of referral option	No impact	56.44	63.77	66.10	0.536
	Medium impact	29.70	27.54	23.73	
	High impact	13.86	8.70	10.17	

21–25 y/o		0–2 topics (*N* = 128)	3–5 topics (*N* = 142)	6–8 topics (*N* = 92)	*P* value
Gender	Female	29.69	42.25	51.09	0.005
Age (y/o)	<40	6.25	14.79	10.87	0.055
	40–49	26.56	31.69	21.74	
	>50	67.19	53.52	67.39	
Speciality	Paediatrician	0.78	2.11	0.00	0.369
	GP	75.00	76.76	70.65	
	Other	24.22	21.13	29.35	
Experience	Inexperienced	31.25	36.62	21.74	0.000
	Experienced	50.78	38.73	33.70	
	Very experienced	17.97	24.65	44.57	
Confidentiality	Discussed	20.31	38.03	68.48	0.000
Prioritization	No impact	22.22	20.00	32.22	0.144
	Medium impact	23.02	27.14	27.78	
	High impact	54.76	52.86	40.00	
Consultation time	No impact	28.57	26.43	38.89	0.295
	Medium impact	27.78	25.71	20.00	
	High impact	43.65	47.86	41.11	
Patient compliance	No impact	52.38	40.00	43.33	0.173
	Medium impact	32.54	35.71	31.11	
	High impact	15.08	24.29	25.56	
Reimbursement	No impact	72.22	65.71	67.78	0.778
	Medium impact	19.05	23.57	20.00	
	High impact	8.73	10.71	12.22	
Lack of skills	No impact	49.21	40.00	58.89	0.047
	Medium impact	34.13	45.00	32.22	
	High impact	16.67	15.00	8.89	
Lack of referral option	No impact	61.90	54.29	71.11	0.151
	Medium impact	26.19	31.43	21.11	
	High impact	11.90	14.29	7.78	

Through the multivariate analysis (Table 3), the first model showed that for all age groups, having discussed confidentiality was significantly related to the number of items screened for, with the likelihood of discussing six or more items increasing by over 3-fold (RRR 5.56, 4.01, and 3.54 for each age group, respectively).

**Table 3.** Results of logistic regression analysis; using the 3–5 HEEADSSS items group as the reference category, by youth’s age group—4 models, sample of 465 physicians located in the French speaking part of Switzerland in 2018.

		10–14 y/o		15–20 y/o		21–25 y/o	
		Low screeners	High screeners	Low screeners	High screeners	Low screeners	High screeners
Model 1 (RRR(95% CI))							
Confidentiality (discussed)		0.22 (0.10–0.51)***	5.56 (2.61–11.9)***	0.28 (0.16–0.49)***	4.01 (2.19–6.32)***	0.42 (0.24–0.72)*	3.54 (2.03–6.17)***
Model 2 (RRR (95% CI))							
Confidentiality (discussed)		0.26 (0.11–0.61)*	4.92 (2.28–10.62)***	0.30(0.17–0.53)***	3.34 (1.79–6.19)***	0.41 (0.23–0.72)*	3.12 (1.77–5.50)***
Experience level	Experienced	0.59 (0.23–1.46)	2.16 (0.24–19.2)	1.24 (0.64**–**2.43)	1.67(0.72**–**3.89)	1.65 (0.94**–**2.87)	1.30 (0.65**–**2.61)
	Very experienced	0.25 (0.09–0.57)*	4.43 (0.52–37.5)	0.68 (0.32**–**1.47)	2.89 (1.26–6.65)*	1.01 (0.51**–**2.0)	2.36 (1.16–4.81)*
Model 3 (RRR (95% CI))							
Confidentiality (discussed)		0.18 (0.07–0.45) ***	**5.67 (2.52**–12.74) ***	0.29 (0.16–0.51)***	**3.21 (1.71**–6.01)***	0.38 (0.22–0.68)**	**3.18 (1.79**–5.66)***
Experience level	Experienced	0.98 (0.35–2.74)	2.14 (0.23–20.07)	1.51 (0.76**–**3.04)	1.48 (0.62–3.51)	1.78 (1.01**–**3.14)	1.29 (0.64–2.60)
	Very experienced	0.40 (0.13–1.22)	4.74 (0.53–42.27)	0.80 (0.36**–**1.79)	**2.50 (1.06**–5.92)*	1.02 (0.51–2.08)	**2.35 (1.15**–4.18)*
Gender	Male	2.11 (0.99–4.52)	0.56 (0.26–1.18)	2.26 (1.27–4.03)*	**0.58 (0.34**–0.98)*	**1.90 (1.13**–3.21)*	0.67 (0.39–1.18)
Specialty	GP	8.46 (3.11–22.9)***	0.72 (0.31–1.63)	1.92 (0.77**–**4.81)	0.61 (0.32–1.17)	N/A	N/A
	Other	5.97 (1.77–20.11)*	0.56 (0.19–1.63)	2.96(1.03–8.54)*	0.72 (0.33–1.59)	0.69 (0.38–1.25)	1.01 (0.53–1.91)
Model 4 (RRR (95% CI))							
Confidentiality (discussed)		**0.84 (0.07**–0.46)***	**5.28 (2.35**–11.87)***	**0.28 (0.16**–0.51)***	**2.99 (1.59**–5.65)**	**0.40 (0.23**–0.72)**	**3.31 (1.84**–5.94)***
Experience level	Experienced	0.86 (0.29–2.56)	2.08 (0.21–20.22)	1.65 (0.80–3.41)	1.37 (0.57–3.28)	1.68 (0.93–3.05)	1.16 (0.56–2.40)
	Very experienced	**0.26 (0.74**–0.93)*	4.87 (0.48–48.93)	0.73 (0.31–1.73)	1.96 (0.78–4.91)	0.86 (0.41–1.81)	1.82 (0.83–3.99)
Gender	Male	1.93 (0.89–4.21)	0.55 (0.25–1.18)	**2.33 (1.30**–4.19)*	0.59 (0.35–1.01)	**1.96 (1.16**–3.33)*	0.68 (0.39–1.21)
Specialty	GP	**9.65 (3.27**–28.5)***	0.75 (0.32–1.72)	2.21 (0.83–5.89)	0.65 (0.34–1.26)	N/A	N/A
	Other	**7.21 (1.94**–26.73)**	0.60 (0.21–1.75)	**3.30 (1.07**–10.17)*	0.73 (0.33–1.62)	0.71 (0.38–1.30)	1.00 (0.51–1.93)
Lack of skills	Medium impact	0.61 (0.25–1.51)	1.44 (0.57–3.60)	0.59 (0.32–1.12)	0.75 (0.41–1.39)	**0.53 (0.30**–0.94)*	0.69 (0.36–1.34)
	High impact	0.55 (0.15–1.99)	0.61 (0.12–3.23)	1.21 (0.51–2.85)	0.48 (0.16–1.37)	0.87 (0.40–1.89)	0.64 (0.24–1.71)

Boldface indicates significant results (*P < 0.05 **P < 0.001 ***P < 0.0001).

N/A: non applicable (cf ‘Methods’ section).

In the second model, with the addition of the physician’s experience, physicians who reported being very experienced regarding adolescent patients and their specific health issues had a more than 2-fold increase in their likelihood of being high screeners regarding the 15–20 y/o (RRR 2.89) and the 21–25 y/o (RRR 2.36) age groups. Confidentiality remained a significant predictor throughout all age groups.

In the third model, when adding significant sociodemographic variables (gender and specialty); confidentiality and physician’s experience remained the main significant predictors. Regarding specialty, GPs’ and physicians from another specialty, were more likely to screen for less than three topics regarding the 10–14 y/o age group (RRR 8.46 and 5.97 respectively). A male physician was more likely to screen for less than three items in age groups 15–20 and 21–25 y/o (RRR 2.26 and 1.90, respectively).

In the fourth model, with the barrier related to a lack of skills being factored in for age groups 15–20 and 21–25 y/o, gender and confidentiality remained significant predictors. Physicians who rated the lack of skills barrier as important were less likely to be low screeners (RRR 0.53).

## Discussion

This study examined physician’s self-reported screening habits regarding youth risk behaviors and social-environmental elements. Findings suggest that most physicians screen for at least 3 elements, the most prevalent topics discussed being home, education and activities, which follow the recommendations for brief risk behaviour screening.^22^ It is important to note that these results do not fully represent physicians’ habits since they are focused on preventive screening habits and do not include secondary or targeted screening.

As previously postulated,^23^ discussing confidentiality seems to be the keystone variable in all risk behaviour screening discussions. This has previously been attributed to confidentiality serving as a discussion opener, enabling youths to feel at ease and, therefore, increasing the possibility of confiding in healthcare providers.^24^ Whether it can be attributed to youths disclosing sensitive topics, after confidentiality is verbally assured, or to physicians who address confidentiality being better versed in sensitive topic discussion methods cannot be determined from this study.

Male physicians have reported a lower number of screened items. This gender influence has previously been established,^25–27^ with female physicians being more likely to screen as well as partake in early prevention. This may reflect patients’ preferences regarding their physician’s gender, as well as physicians’ communication styles.^27^ Drawing male PCP’ attention to this point during adolescent health training may improve their screening techniques and habits.

Previously established barriers to screening,^16^ especially the lack of consultation time and prioritization issues, have been reported by physicians as having an moderate to high impact on their screening abilities. However, most of these barriers have not been found to have a statistically significant impact regarding screening habits within this sample. This could be attributed to two psychological elements. First, since the screening process does not appear hindered, the analysed barriers could be cognitive distortions rather than the actual limiting factors. Secondly, it could be attributed to the social desirability bias, with physicians reporting on external barriers rather than reflecting on the presence of internal ones.

Self-reported skill set and feeling of experience can be constructed as reflections of self-efficacy (the belief that one can be successful while carrying out a task). Through this study, we found out that physicians who reported feeling experienced regarding adolescent health addressed a higher number of topics with youths. Moreover, the barrier related to a lack of skill set regarding adolescent health care was found to impact screening rates despite it being rated as having a low impact by most physicians.

It would, therefore, seem that the actual barriers are not yet fully cognized by physicians. This could indicate that while the improvement of the barriers previously put forward^16–18^ is important, increasing physician’s self-efficacy is indispensable. These findings corroborate previous studies^28^ on the topic of self-efficacy.

This study’s strengths are the diversity of physicians reached. Its limitations are its cross-sectional design, which prevents any causality relation. In addition, as questionnaires were anonymous, a social desirability bias cannot be excluded. Moreover, Physicians were asked whether barriers were important, but not whether they were able to overcome them and how, which could be a topic for further research.

## Conclusion

This study demonstrates that most physicians screen youths preventively for at least three risk behaviours. Barriers such as lack of consultation time and prioritization issues were found to be critical according to physicians but did not hinder screening habits. The main element impacting screening habits put forward through this study was assuring confidentiality. An interesting element is self-efficacy, with physicians who feel lacking the necessary skill set screening less. Therefore, improving physicians’ self-efficacy during training could be a useful tool to improve screening habits.

Further studies should focus on identifying the levers and obstacles to strengthen physician’s self-efficacy as well as identifying further barriers to screening and tools to overcome them.

## Supplementary Material

cmad106_suppl_Supplementary_Checklist

## Data Availability

The data underlying this article will be shared on reasonable request to the corresponding author.
